# How distinct are sleep sites from active sites across habitat types in lizards?

**DOI:** 10.1007/s00265-026-03693-w

**Published:** 2026-01-15

**Authors:** Nitya Prakash Mohanty, Anbazhagan Abinesh, Saumitra Dhere, Maria Thaker

**Affiliations:** 1 Centre for Ecological Sciences, Indian Institute of Science, Bengaluru, India; 2 Département Adaptations du vivant, UMR 7179 CNRS/ Muséum National d’Histoire Naturelle, 57 rue Cuvier, 75005 Paris, France

**Keywords:** Site selection, Diel shift, Diurnal-nocturnal, Reptile, Agamidae, Perch

## Abstract

Sleep poses constraints, such as increased vulnerability to predators, that can lead to differences in the use of habitat components across the diel cycle. However, very few studies have systematically evaluated site use of animals across both active and sleep phases. We quantified site use during the active and sleep phases in 412 individuals of eight species (six genera) of agamid lizards, from four habitat types (arboreal, semi-arboreal, rupicolous and ground). Sleep sites differed for five out of the eight species, in all habitat types except rupicolous. Semi-arboreal and arboreal lizards slept on narrower and more compliant perches, likely to detect and avoid predators, whereas ground-dwelling lizards slept in sheltered sites that probably afford a stable thermo-hydric environment. For most species, sleep sites differed from active sites in only a few characteristics, indicating that lizards respond to the potential costs of sleep while being constrained by their morpho-functional abilities. We found equivocal pattens of lower variability of sleep sites compared to active sites, which may be due to low variability in perch options or low costs of sleep in some habitat types. This study provides a large-scale test of diel shifts in habitat use and highlights the need to integrate sleep microhabitat use into ecological and conservation research.

## Introduction

Sleep is a large part of an animal’s life, and yet the behavioural ecology of sleep in the wild is poorly understood for many taxa (Rößler and Klein 2024). When and for how long an animal sleeps influences its physical and cognitive wake performance (Tougeron and Abram 2017; Johnsson et al. 2022) and ultimately, fitness (Lesku and Rattenborg 2022). Where an animal sleeps is just as important. Sleep, as a physiological state, imposes restrictions on the required thermal-hydric conditions (e.g., core body temperature needs to drop to induce sleep in mammals; Harding et al. 2019). As a behavioural state, sleep reduces an individual’s environmental awareness and is coupled with increased response time to stimuli, thereby elevating the risk of predation (Lima et al. 2005). Predation risk during the sleep phase may be even more challenging to avoid if the predator community shifts with the diel cycle (Clark et al. 2003). Therefore, selection of suitable sleep sites is critical as these sites could reduce predation risk (Hamilton 1982; Jucá et al. 2020; but see Bidner et al. 2018), as well as aid in postural stability and thermal comfort (Samson and Hunt 2012, 2014). Despite its importance for survival, information about sleep site selection is missing for many groups of animals, as are structural and functional comparisons of sleep and active sites.

In response to the distinct requirements during sleep compared to the active phase, animals may use different components of their habitat across diel phases (Elliott 2005; see Freiberg 2020). Many taxa, including primates (Caselli et al. 2017; Jucá et al. 2020), waterbirds (Jirinec et al. 2016), and lizards (Singhal et al. 2007) have divergent sleep and wake site use. These differences entail using dissimilar substrates (ground vs. tree) or parts of the same substrate that vary in position, dimension, or compliance. For example, in *Alouatta* monkeys, nocturnal sleep sites on trees are taller and further away from the main trunk than diurnal sites (Jucá et al. 2020). In reptiles, the use of narrow and compliant perches for sleep has been observed in snakes and several lizards (reviewed in Mohanty et al. 2022), and these provide the purported benefit of exclusion or early detection of any climbing predators through tactile cues (Anderson 1998). In all these cases, perceived safety of sleep sites can affect sleep architecture (Tisdale et al. 2018).

Agamid lizards (Family Agamidae), a highly speciose group (600 species) with remarkable diversification into most terrestrial habitats (Uetz et al. 2024), present a good system to test the generality of sleep site specialization across taxa and habitat types. The opportunity to select distinct sleep sites is dependent on the available variation in substrate types and their dimensions, which could differ between habitat types. Further, species which are morpho-functionally able to exploit different strata, such as semi-arboreal lizards that can use both ground and vegetation, have a range of choices that can match their requirements for the active and sleep phases. Some semi-arboreal agamid lizards indeed use distinct sleep sites in understory vegetation that are thin and compliant whereas tree trunks are used as active sites (*Coryphophylax subcristatus, C. brevicaudus*; Mohanty et al. 2016). Similarly, *Monilesaurus rouxii* lizards use tree trunks during their active phase but sleep in the understory, on higher, thinner, and more horizontal perches that are farther from the main trunk (Bors et al. 2020). On the contrary, the rock-dwelling (hereafter, rupicolous) agamid *Psammophilus dorsalis* appears to use similar perches on rocks during both day and night, although a quantitative comparison across diel phases is missing (Mohanty et al. 2021). Sleep sites of ground-dwelling and fully arboreal agamid lizards remain undescribed.

In this study, we aim to quantify the differences between active and sleep sites using eight species of agamid lizards belonging to six genera, across four major habitat types (arboreal, semi-arboreal, rupicolous, and ground). Across habitat types, sleep sites could be nested within active sites, diverge partially or even fully. We expect sleep site characteristics to be less variable than active sites if only specific types of perches provide safety or stability during sleep; or if the active phase requires a wider range of sites for different activities. By quantifying the structural differences between sleep and active sites, this study provides an empirical test of intra-diel microhabitat discordance at a large scale and discusses the likely benefits and consequences of sleep site selection.

## Methods

**Fig. 1:**
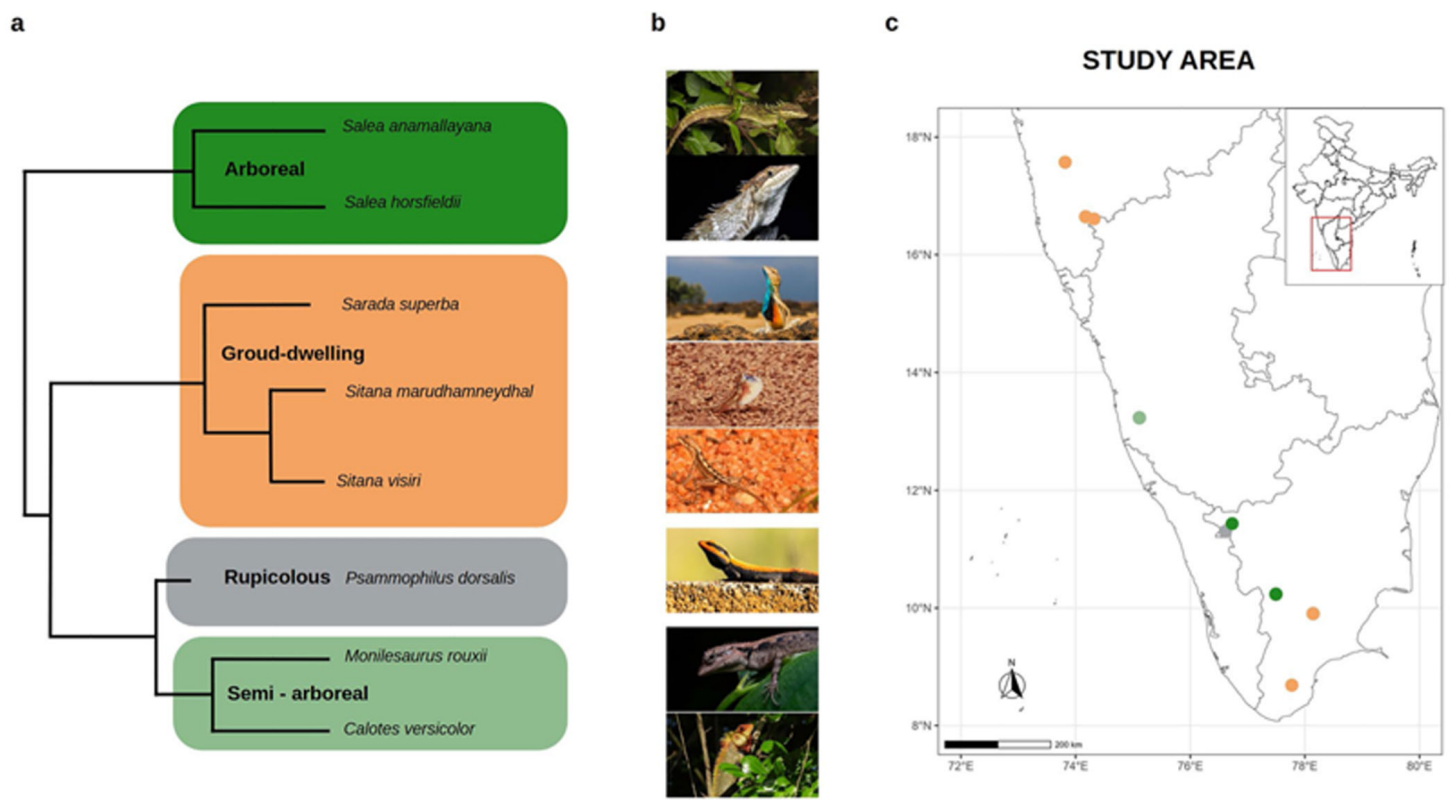
**a**) Phylogenetic relationships, **b**) in-situ photographs (credit: inaturalist.org), and **c**) sampling locations of eight species of Agamid lizards of four habitat types, in India

We carried out the study from April 2021 to May 2022 in peninsular India (Fig. 1). We initially targeted 10 species but only eight species could be sufficiently sampled for active and sleep sites (excluding *Sarada darwini* and *Sitana laticeps*). These species were chosen to cover a range of habitats: arboreal (*Salea horsfieldii, Salea anamalayana*), semi-arboreal (*Calotes versicolor, Monilesaurus rouxii*), ground (*Sitana visiri, Sitana marudhamneydhal, Sarada superba*), and rupicolous (*Psammophilus dorsalis*). All these species belong to the subfamily Draconinae and are closely related to each other overall (Grismer et al. 2016; Pal et al. 2018). At a finer phylogenetic scale, *Monilesaurus* and *Psammophilus* are sister genera and together, are sister to the genus *Calotes* (Pal et al. 2018); *Sitana* and *Sarada* are also sister genera to each other (Deepak et al. 2016). Habitat types of the sampled species are tightly linked to the fine-scaled pattern of phylogeny (Fig. 1), with both arboreal species coming from the same genus, both semi-arboreal species from sister genera and all three ground-dwelling species from sister genera. Apart from differences in habitat, all species share broad similarities in ecology, as they are diurnal, heliothermic, and insectivorous.

We chose sampling sites to be representative of typical habitats of the species and devoid of direct human disturbance (Online Resource 1 Table 1). The only exceptions were the sampling sites for *Salea* spp., which were transformed from native forests to Eucalyptus and Acacia plantations adjoining Shola-grasslands. Day sampling spanned from 0700 h to 1100 h, and night sampling from 1900 h to 2300 h, the timing of which was informed by previous studies on these and other species of diurnal agamid lizards (Radder et al. 2005; Mohanty et al. 2016, 2021). Two to three researchers searched with equal attention to all habitat strata, from the ground up to 4 m; this approach was particularly important in the absence of a priori expectations for most species and to avoid bias. The sampling protocol broadly followed previous studies on sleep sites of semi-arboreal species (Mohanty et al. 2016; Bors et al. 2020) and rupicolous species (Mohanty et al. 2021). We equated nocturnal perch sites to sleep sites, as individuals fulfilled some criteria for behavioural sleep at night (Piéron 1912; Tobler 1995): prolonged inactivity, relatively low responsiveness (e.g., easy to approach and capture by hand, without evoking movement), and typical sleep postures (head resting on perch, eyes closed).

For lizards of all habitat types, we categorised the substrate type (e.g., shrub, tree, rock), perch type (e.g., leaf, branch), perch angle (e.g., horizontal, angular, or vertical), and recorded perch height (perpendicular distance from lizard’s head to ground). For lizards observed on plants, we also measured the distance to main trunk (non-linear distance along branches), perch diameter (branch circumference at or closest to the location of lizard’s head), and trunk girth (maximum girth of trunk). Compliance of a perch is generally calculated using standard weights and measuring the resulting displacement (Samson and Hunt 2014), but diameter is a good proxy of compliance for branches and trunks (Gilman and Irschick 2013). We also recorded ‘head direction’ with respect to the potential approach path of a climbing predator as ‘inward’, ‘outward’, or ‘perpendicular’ (following Mohanty et al. 2016). For lizards on rocks, we defined head direction as ‘upward’, ‘angular-upward’, ‘perpendicular’, ‘downward’, or ‘angular-downward’ (the latter case was never observed). Individuals observed on the ground were not assigned any head direction.

As we were interested in the site use contrast between active and sleep phase in the same habitat, we did not estimate site selection by quantifying availability. Further, we did not sample the thermal-hydric conditions as an axis of habitat, as we considered it meaningful only in the presence of large spatial-scale capturing of availability, which we were unable to collect. We marked individuals using a felt pen on capture and did not re-collect habitat information for any individuals (i.e., different individuals were sampled for active sites and for sleep sites). As our study involved focal animals in the field, it was not possible to record data blind.

### Data analyses

We retained observations of only adults with complete information in the dataset, excluding juveniles as they had limited sample sizes. We followed the protocol for data exploration by Zuur et al. (2010). We identified and excluded outliers (*n* = 21) by visualizing the raw data and subsequently excluded them by Tukey’s two fence method (values beyond the interval between Quartile 1 − 1.5x Interquartile range and Quartile 3 + 1.5x Interquartile range). Data was screened for multicollinearity by plotting pairwise scatterplots and examining correlation coefficients. We did not pool data at the level of habitat as preliminary results showed distinct patterns between species within habitat types (e.g., *Calotes versicolor* and *Monilesaurus rouxii*). Although we sampled in a range of habitats, species within each habitat were closely related, precluding analysis of phylogenetic signals. All statistical analyses were performed using R version 4.4.1 (R Development Core Team 2024).

We compared microhabitat use across active and sleep phases on two scales: at a broad scale of parameter space composed of all variables and at a fine scale of each perch characteristic. To examine the overall parameter space across diel phase, we ran one principal component analysis (PCA) for each species. Continuous variables (perch height, trunk girth, perch diameter, and distance to trunk) were standardized and categorical variables (substrate type, perch angle, and head direction) were dummy coded (i.e., each category of a variable was defined as a new variable and scored as 0 (no) or 1 (yes); Bertolo et al. 2012; Miró et al. 2017). We did not include perch type in the PCAs, as we considered substrate type to be an adequate categorical descriptor at this scale and to avoid including a large number of perch types that are possible across substrate types. The PCAs for arboreal and semi-arboreal species included trunk girth, perch diameter, and distance to trunk, in addition to substrate type, perch height, perch angle, and head direction. For ground-dwelling and rupicolous species, PCAs included substrate type, perch height, perch angle, and head direction. However, for all three ground-dwelling species these variables had limited to no variation within or across diel phases, i.e., substrate type was predominantly ‘ground’, perch height was 0 mm, perch angle was ‘horizontal’. Thus, a PCA could not be run for ground-dwelling *Sitana marudhamneydhal*; for *Sarada superba* and *Sitana visiri*, most observations coincided on one or two points on the PCA in the sleep phase, precluding any demarcation of parameter space by ellipses (see below).

We overlaid observations on the first two components of the PCA (PC1 and PC2) for each species and tested if they differed in their relative positions and variance using Permutational Multivariate Analysis of Variance (PERMANOVA; with 999 permutations) and multivariate homogeneity of groups dispersions, respectively. We then constructed multivariate normal ellipses (at 95% confidence interval) on these observations for each diel phase in all species, except the three ground-dwelling species which had limited variability. For these pairs of ellipses, we computed area (breadth), overlap, and uniqueness in absolute and in percentage of the total parameter space (union of ellipses of both diel phases).

At the fine scale, we compared each perch characteristic across diel phase (active vs. sleep) at the species-level. Observation of an individual at a specific perch location is the outcome of a series of decisions, starting from choosing a substrate among several available substrates of varying girth, to climbing a certain distance from the ground and moving away from the trunk, and finally to orienting the head in a particular direction. Therefore, we examined the differences across diel phase (‘active’, ‘sleep’) in each perch characteristic individually. We used chi-square test of proportions for categorical variables (substrate type, perch type, perch angle, and head direction) and Ordinary Least Squares regressions (OLS) for continuous response variables (i.e., perch structure measurements). Substrate types and perch types were reclassified prior to analysis to reduce the number of categories; for example, saplings were merged along with shrubs and observations with a combination of perch types were assigned to one type (e.g., branchleaf to leaf). For OLS regressions, all perch measurements were log transformed (1 + log_10_); normality and homogeneity of variance of residuals, as well as model validity were checked using diagnostic plots (Zuur and Ieno 2016) along with Levene’s test. As trunk girth and perch diameter were highly correlated across species (*r* > 0.5), we considered only trunk girth and not perch diameter in subsequent models. Models with trunk girth as the response variable had phase as the only predictor. For models of perch height and distance to trunk in semi-arboreal and arboreal species, we included both phase and trunk girth as predictors because trunk girth can influence other perch measures (following Mohanty et al. 2016). In the case of rupiculous and ground-dwelling species, models for perch height had only phase as the predictor. We did not include sex as a predictor as we considered our sample sizes too low for testing sex effects by phase in each species. Body size was similar for samples of both phases in all species (Online Resource 1 Table 1) and therefore was not included in the analyses as a covariate.

## Results

**Fig. 2:**
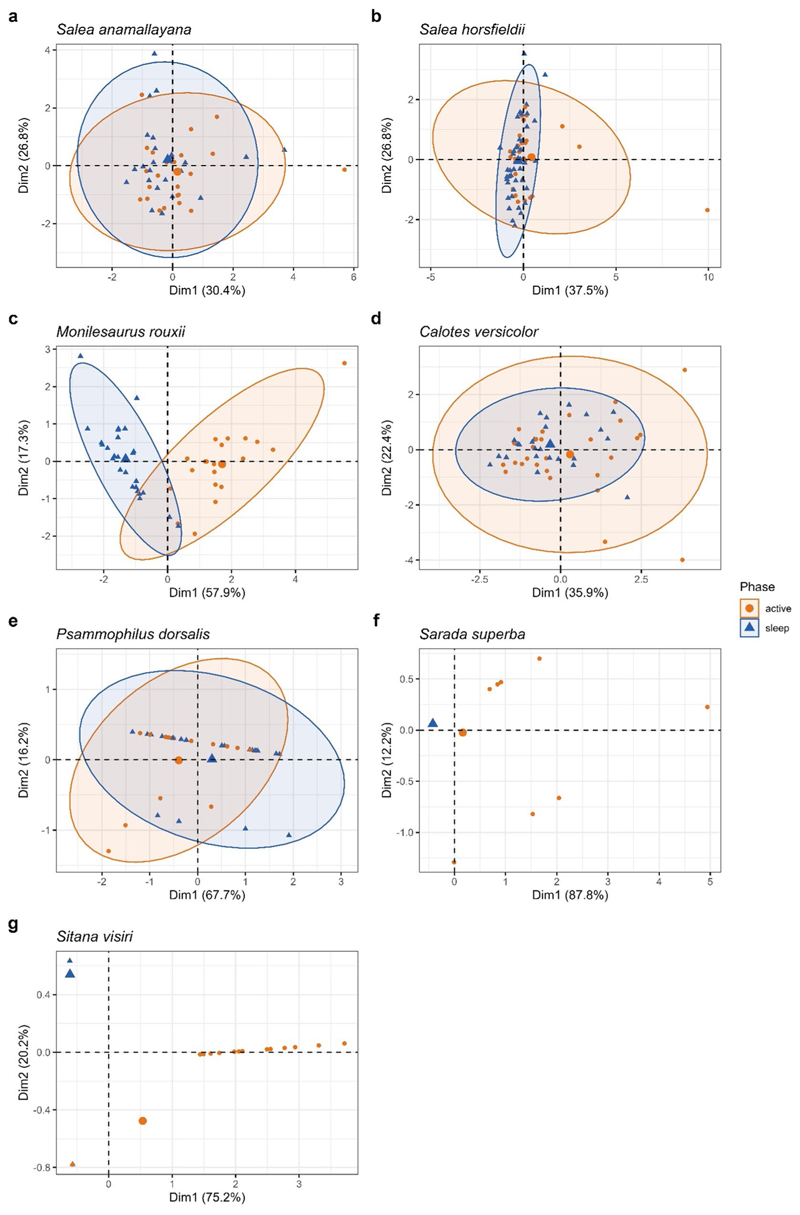
Parameter spaces of Agamid lizards in active and sleep phases, derived from principal component analysis (with 95% confidence interval ellipses), inputted with perch characteristics (substrate type, perch height, perch angle, trunk girth, perch diameter, distance to trunk) and their use by individuals (head direction). PCAs for *Psammophilus dorsalis, Sarada superba*, and *Sitana visiri* are based on substrate type, perch height, perch angle, and head direction. Larger points/triangles denote centroids

We measured site use in eight species of agamid lizards, in the active and sleep phases, for 210 and 212 individuals respectively (Online Resource 1 Table 1). We generated a parameter space, constituted from PC1 and PC2, for all species except the ground-dwelling *S. marudhamneydhal* (Fig. 2). For arboreal *Salea* spp., PC1 represented structural robustness with trunk girth and perch diameter loading positively, and PC2 represented distance from ground with positive loadings of perch height and distance to trunk (Fig. 2a, b; Online Resource 1 Table 2). For semi-arboreal *M. rouxii* (Fig. 2c), PC1 was a combination of structural robustness, perch angle (vertical), and head direction (out-ward), whereas PC2 comprised distance from ground with indirect effects of structural robustness, and substrate type (tree). The other semi-arboreal species *C. versicolor* (Fig. 2d) had a combination of structural robustness and substrate type (tree) associated with PC1 and distance from ground with PC2. In the case of rupicolous *P. dorsalis* (Fig. 2e), PC1 and PC2 represented perch height and head direction (upward) respectively. For ground-dwelling *S. superba*, PC1 denoted perch height and PC2 denoted substrate type (ground), whereas for *S. visiri*, PC1 was a combination of perch height, substrate type (shrub), and head direction (outward) and PC2 comprised substrate type (ground-under shrub).

The multivariate normal ellipses of sleep sites and active sites overlapped to varying degrees (Table 1). Semi-arboreal *M. rouxii* showed little overlap between phases (10.65%; PERMANOVA: *p =* 0.001; R^2^ = 0.56), whereas arboreal *S. anamallayana* displayed high overlap (73.12%; PER-MANOVA: *p =* 0.344; R^2^ = 0.02). Although PERMANOVA test on PC1 and PC2 significantly differed between diel phases for arboreal *S. horsfieldii* (overlap: 21.16%; *p =* 0.036; R^2^ = 0.04) and rupicolous *P. dorsalis* (overlap: 56.00%; *p =* 0.046; R^2^ = 0.09), the associated model performances were poor (R^2^ < 0.1). Parameter space for the semi-arboreal *C. versicolor* did not differ between phases (overlap: 38.98%; *p =* 0.137; R^2^ = 0.03). In the case of ground-dwelling lizards, observations along PC1 and PC2, diverged between phases for *S. visiri* (*p =* 0.001; R^2^ = 0.31) but not for *S. superba* (*p =* 0.11; R^2^ = 0.05). Variance within phase in observations along PC1 and PC2 was significantly different only in the case of *S. visiri* (greater variation during active phase compared to sleep phase; *p =* 0.002). We found tendential differences for *C. versicolor* (greater variation during active phase; *p =* 0.064) and *S. superba* (greater variation during active phase; *p =* 0.073).

At the level of site characteristics, sleep and active phases differed in lizards of most habitat types, except in the ground-dwelling *S. superba* and *S. marudhamneydhal* and rupicolous *P. dorsalis*. Both species of semi-arboreal lizards (*C. versicolor, M. rouxii*) shifted their substrate types from trees during the active phase (66.7% of lizards) to shrubs during sleep (71.5% of lizards). The ground-dwelling *S. visiri* slept on the ‘ground under vegetation’ in contrast to their use of shrubs and rocks during the active phase (Table 3; Online Resource 1 Fig. 1). Within substrates, perch types used for sleep differed from those used during the active phase in arboreal *S. anamallayana* (greater use of leaves), semi-arboreal *C. versicolor* and *M. rouxii* (greater use of branches and leaves), and terrestrial *S. visiri* (greater use of sandy soil; Table 3).

**Fig. 3:**
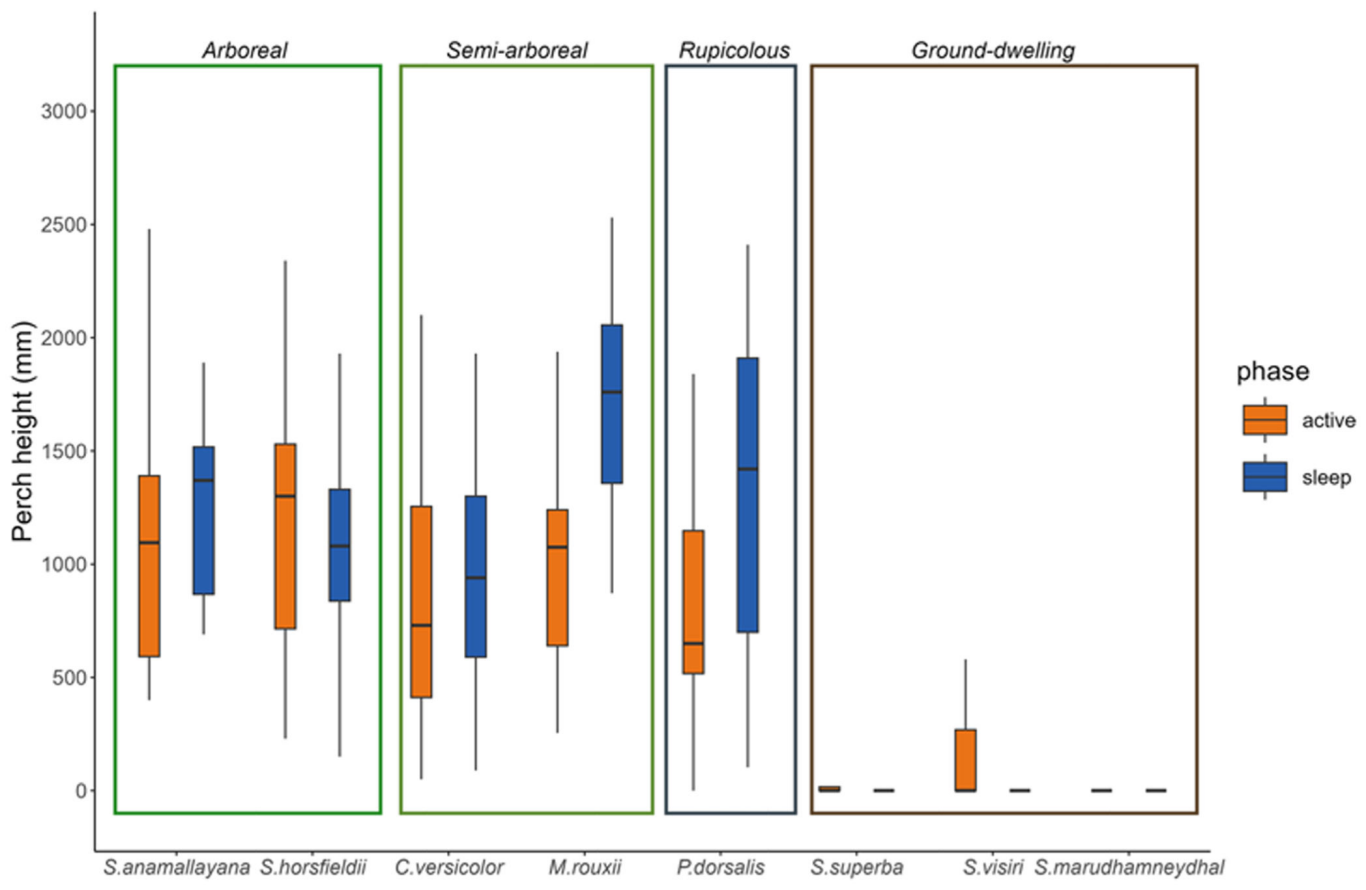
Perch heights of Agamid lizards of four habitat types, during active and sleep phases

Perch height did not differ between phases for most species, except in semi-arboreal *M. rouxii*, which used higher perches during sleep and ground-dwelling *S. visiri*, which used lower perches during sleep (Fig. 3; Table 2). The arboreal *S. anamallayana* and semi-arboreal *C. versicolor* also used higher perches during sleep compared to their active phase, but model performance for these comparisons was poor (R^2^ < 0.1; Table 2). Most species used similar perch angles during both phases (e.g., vertical for rupicolous *P. dorsalis*; Table 3), except *M. rouxii* which used more angular sleep perches as compared to vertical perches during the active phase (χ^2^ = 33.7, *p* < 0.001) and *S. visiri* which used more horizontal sleep perches against vertical perches during the active phase (χ^2^ = 14.39, *p* < 0.001). *S. horsfieldii* showed a similar pattern of sleeping more frequently on horizontal perches, but this difference between phases was tendential (χ^2^ = 5.42, *p* < 0.066).

**Fig. 4:**
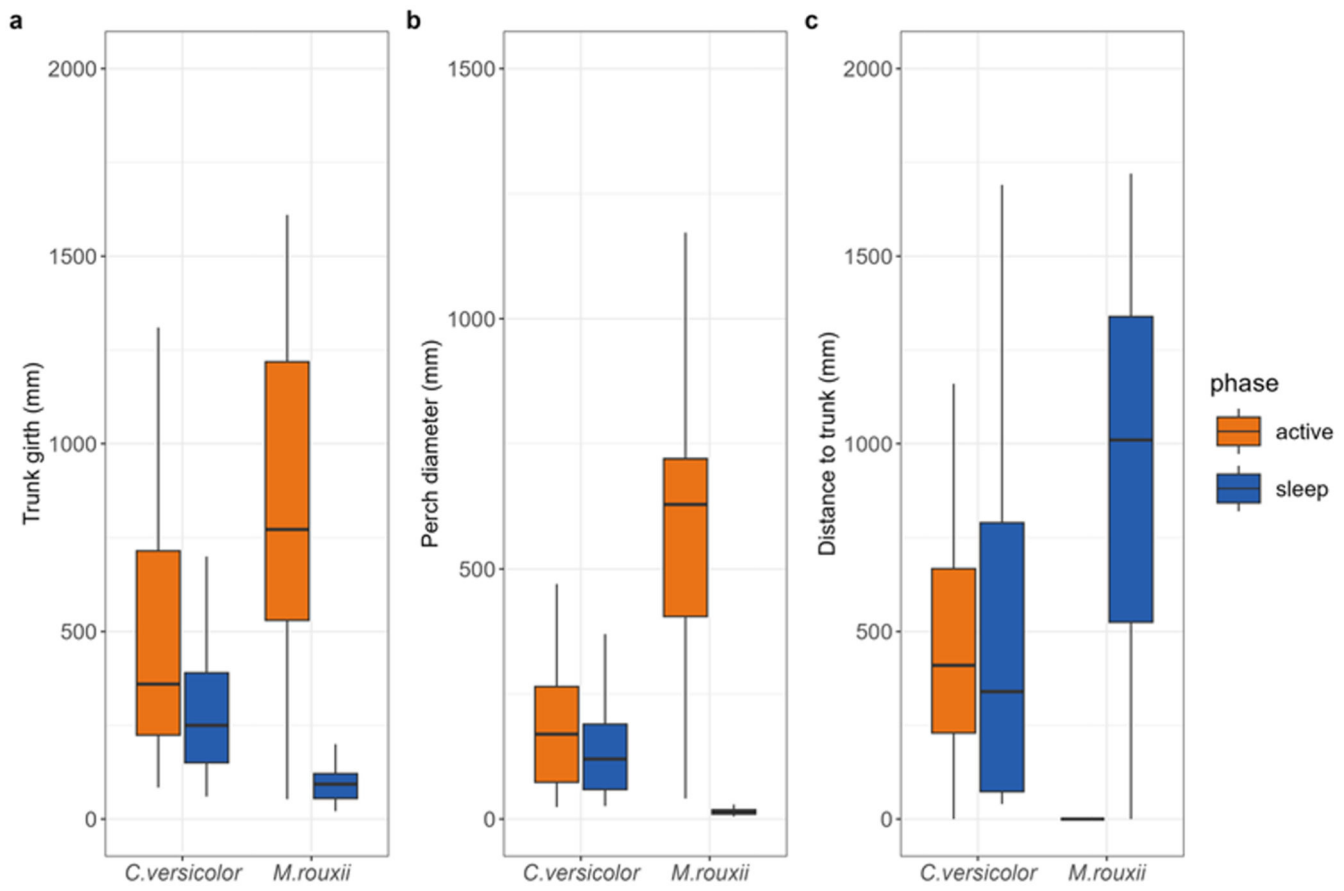
Perch characteristics of semi-arboreal lizards *Calotes versicolor* and *Monilesaurus rouxii* during active and sleep phases

Most arboreal and semi-arboreal species used plants of narrower trunk girth during the sleep phase, except *S. anamallayana* (Table 2). Although *C. versicolor* also slept on narrower trunks, the variance explained by the corresponding model was low (R^2^ < 0.1; Table 2; Fig. 4). After accounting for trunk girth, perch diameter was narrower and distance to trunk was greater during sleep, only in the case of *M. rouxii* (Fig. 4; Table 2). For semi-arboreal but not for arboreal lizards, head direction was more ‘inwards’ than ‘outwards’ or ‘perpendicular’ (*C. versicolor*: χ^2^ = 13.3, *p* < 0.001; *M. rouxii*: χ^2^ = 30.57, *p* < 0.001; Online Resource 1 Table 3) during the sleep phase compared to the active phase. The rupicolous *P. dorsalis* had mostly ‘upward’ head direction in both phases (χ^2^ = 4.95, *p =* 0.174).

## Discussion

Sleep and activity impose different ecological requirements and therefore, sites used by animals in these two phases could diverge to meet these needs. We tested the generality of the diel shift in site use in eight species of agamid lizards occurring in four habitat types, and found support for differences in sleep site characteristics in five out of the eight species. Lizards from arboreal, semi-arboreal, and ground habitats, but not from a rupicolous habitat differed in specific characteristics of sites used during the active and sleep phases. However, when site characteristics were combined into a single parameter space, the overlap between diel phases varied across species. Sleep site characteristics were not consistently less variable than those of active sites, with only one ground-dwelling species showing a significant difference. This study is one of the first to test the potential discordance in microhabitat requirements between diel phases across multiple species. We also provide new data on sleep sites for six of the eight species considered.

At a broad scale with all substrate and perch characteristics combined into a parameter space, we found considerable overlap between diel phases, with limited uniqueness for each phase (Table 1; Fig. 2). Only semi-arboreal *M. rouxii* and ground-dwelling *S. visiri* showed clearly diverging diel patterns in the parameter space. Although PERMANOVA tests significantly differed between phases for arboreal *S. hosfieldii* and rupicolous *P. dorsalis*, these differences are likely weak given the poor model performances. These results broadly indicate that most lizards do not require sleep sites that differ along multiple axes. We find limited support for the expectation of low variability of sleep site characteristics with a significant difference only in case of *S. visiri* (Table 1). However, a similar but not significant trend of low variability sleep sites is apparent for another ground-dwelling species (*S. superba*) and the semi-arboreal *C. versicolor*. This contrasts with no differences for the other five species. This could mean that a range of perch sites could provide the requirements of safety, stability, or microclimatic buffering during sleep. Comparable variability in perch characteristics could also be a result of limited variation in available perches or similar microclimatic requirements and predation risk during both sleep and activity in some species (Shiratsuru et al. 2023). Age-sex differences in perch requirements during active phase (Delaney and Warner 2016) and sleep phase (Barnett and Poe 2023) could also contribute to variability (or lack thereof) between diel phases.

In tree-dwelling species of arboreal and semi-arboreal habitats, the use of compliant (flexible) perches for sleep was prevalent. Arboreal *S. anamallayana* used thin plants across diel phases (Table 2), similar to the case of the Cape dwarf chameleon *Bradypodion pumilum* (Tolley 2023), whereas *S. horsfieldii* slept on even thinner plants (34.5 mm in trunk girth). It is notable that both *Salea* spp. used shrubs instead of available trees such as *Eucalyptus* and *Acacia*, though *S. anamallayana* is known to use trees in undisturbed Shola forests (Deepak and Vasuedevan 2008). Semi-arboreal agamids *C. versicolor* and *M. rouxii* showed substantial diel shifts to narrower sleep sites, by choosing new substrates (shrubs instead of trees). The observed use of compliant perches (thin perches on plants of narrow trunk girth) with putative benefits for predator exclusion and detection, is consistent with many tree-dwelling lizards and squamates in general (Mohanty et al. 2022). Therefore, predator avoidance appears to be a substantial criterion for sleep site use in this group. Predation risk is also known to shape sleep behaviour and architecture in mammals (Capellini et al. 2008; Pozzi et al. 2022) and birds (Tisdale et al. 2018; Ferretti et al. 2019). Direct experimental evidence on the efficacy of compliant perches for quicker detection of predators through vibrational cues still needs to be established in lizards (e.g., Warkentin 2005).

Diel shifts in perch sites were less pronounced in lizards of both ground and rupicolous habitat types. All three ground-dwelling agamids (*S. visiri, S. marudhamneydhal, S. superba*) slept on the ground whereas the rupicolous *P*. dorsalis slept on rocks. Sleeping on the ground, compared to on a higher perch, can be risky because it allows easier access to ground-dwelling predators and less time for detection. Mammals are hypothesized to sleep higher off the ground for this reason (Duarte and Young 2011; Svensson et al. 2018), as are some lizards (e.g., *Brookesia* chameleons; Razafimahatratra et al. 2008). The fact that these species used ground or rock substrates in both active and sleep phases may reflect morphological or performance constraints (Irschick and Losos 1999). Alternately, low variability in available substrate types in these habitats could lead to high but proportionate use of the dominant type (e.g., rocks or ground), but we lack this ecological data. Only *S. visiri* showed a shift in the use of substrate and perch types across diel phases, notably by sleeping on the ‘ground under vegetation’. Sleeping closer to the ground, where humidity is higher, may buffer against thermo-hydric stress (Samson and Hunt 2012). This could be particularly important in open landscapes inhabited by *Sitana* and *Sarada* spp., especially during sleep when behavioural thermoregulation is limited. However, neither *S. marudhamneydhal* nor *S. superba* slept on the ‘ground under vegetation’. A quantitative assessment of thermal-hydric parameters at sleep and active sites for these and other lizards would shed light on perch use decisions (Penado et al. 2015).

We found mixed patterns for the differences in perch height between the active and sleep phases across lizards (Fig. 4). Although most tree-dwelling species such as *M. rouxii* shifted to higher perches for sleep (similar trend in *C. versicolor* and *S. anamallayana* but not statistically significant), most ground-dwelling species such as *S. visiri* moved lower (similar trend in *S. superba* but not statistically significant). Higher sleep sites could aid in reducing predation risk by increasing vertical distance from terrestrial predators and provide opportunities for early basking or territory guarding (Singhal et al. 2007; Mohanty et al. 2022). For ground-dwelling lizards, however, thermo-hydric benefits of sleeping closer to the ground appears more important that being out of reach of predators, a cost they potentially offset by being highly sensitive to ground vibrations (AA pers. obs.).

Perch angles were similar across diel phases for most species, except for the use of more angular perches in *M. rouxii* and more horizontal perches in *S. horsfieldii* (but not statistically significant). This indicates the ability of species to maintain sleep postures on a variety of perches, including the extreme case of vertical sleeping in *P. dorsalis*. The head position of animals at the sleep perch could aid in predator detection. For example, individuals that sleep with their head towards the proximal end of the perch (‘inwards’) as observed in semi-arboreal lizards in this study, could use visual cues to confirm a threat once awakened by vibrations (Mohanty et al. 2022). On the contrary, many lizards sleep with their head towards the distal end of the perch (e.g., *S. hosfieldii*; Ikeuchi et al. 2012; Storks and Leal 2020) which may enable them to escape to an adjoining plant. Head direction may also be unrelated to escape response but rather reflect the direction from which the individual reaches the site (e.g., from the ground or adjoining plant). The role of head direction in escape during the sleep phase is not sub-stantiated; for example, *M. rouxii* lizards, irrespective of ‘inward’ or ‘outward’ head direction, showed similar flight initiation distance and a common strategy of ‘perch release’ (Raxworthy 1991), wherein they drop from the perch and freezing on the ground when disturbed at night (Bors et al. 2022). Escape behaviour during sleep and its relationship with perch characteristics is little understood in lizards (see Losos and Irschick 1996), in contrast to the large body of information on escape behaviours during the active phase (Samia et al. 2016).

The structural niche space occupied by animals in the two diel phases could be influenced by several considerations which were not directly addressed in this study. The availability and variability of perch structures may affect perch selection (Bors et al. 2020), along with inter-specific competition (Singhal et al. 2007; but see, Mohanty et al. 2016 for an example of high overlap of sleep sites in two syntopic species of agamid lizards). Both arboreal lizards in our dataset could not be sampled in undisturbed sites and therefore, the results presented here may not be fully representative. Within available perches, individuals are likely to be constrained to perches on which their functional performance is relatively better, which in turn is linked to morphology (Irschick and Losos 1999; Kohlsdorf et al. 2001; Goodman et al. 2008; Ord and Klomp 2014). If morphology is closely related to the active phase, it may constrain use of sleep sites that differ from active sites. Microhabitat use across diel phases could also differ between males and females, as well as adults and juveniles (Delaney and Warner 2016; Mohanty et al. 2022); however, we were unable to assess this due to limited sample sizes. Furthermore, the type of sleep site may change with season (Ramanankirahina et al. 2012), which is not captured in this ‘snapshot’ study, or between populations (Herrel et al. 2011). Finally, the influence of phylogeny on micro-habitat discordance between diel phases needs to be evaluated with a larger dataset of lizard species. Although sleep sites have long been identified as a key component of an animal’s habitat (Amlaner and Ball 1983; Anderson 1984, 1998), this critical information is well-assessed only in primates (Pozzi et al. 2022) and is missing for most species, including reptiles (sleep site information on less than 3% species; Mohanty et al. 2022). We suggest remedying this knowledge short-fall by documenting sleep sites along with active sites in the wild. In a changing world, with landscape transformation, light pollution, and climate warming, it is pertinent to understand behavioural choices, such as site selection, that promote sleep.

## Supplementary Material

The online version contains supplementary material available at https://doi.org/10.1007/s00265-026-03693-w.

Supplementary Material 1

Supplementary Material 2

## Figures and Tables

**Table 1:** Measures of the relationship between active and sleep phase on parameter spaces constructed from principal component analyses in Agamid lizards. Areas of multivariate normal ellipses (at 95% confidence interval) for each phase are unitless; values in parentheses represent percentages of total area of parameter space. Results of PERMANOVA tests on observations along principal components 1 and 2, with the associated model R^2^ values in parentheses. ‘Variance’ indicates results of multivariate homogeneity of groups dispersions tests. Dashes denote no tests or calculations performed due to lack of variability in data. Statistically significant tests (α = 0.05) in bold

Habitat	Species	Active area	Sleep area	Total area	Active unique	Sleep unique	Overlap	PERMANOVA *p*-value	Variance *p*-value
*Arboreal*	*Salea anamallayana*	26.72	27.84	31.51	3.68 (11.67%)	4.79 (15.21%)	23.04 (73.12%)	0.344 (0.02)	0.831
	*Salea horsfieldii*	36.78	9.84	38.49	28.64 (74.42%)	1.70 (4.42%)	8.14 (21.16%)	**0.035** (0.04)	0.596
*Semi-arboreal*	*Calotes versicolor*	42.29	16.49	42.29	25.81 (61.02%)	0 (0%)	16.49 (38.98%)	0.150 (0.03)	0.064
	*Monilesaurus rouxii*	13.51	7.95	19.39	11.44 (59.00%)	5.88 (30.35%)	2.07 (10.65%)	**0.001** (0.56)	0.555
*Rupicolous*	*Psammophilus dorsalis*	7.73	9.04	10.75	1.70 (15.86%)	3.02 (28.12%)	6.02 (56.00%)	**0.046** (0.09)	0.201
*Ground-dwelling*	*Sarada superba*	-	-	-	-	-	-	0.11 (0.05)	0.073
	*Sitana visiri*	-	-	-	-	-	-	**0.001** (0.31)	**0.002**
	*Sitana marudhamneydal*	-	-	-	-	-	-	**-**	-

**Table 2:** Ordinary least square regressions on perch characteristics of Agamid lizards in relation to diel phase (reference: active phase). Models for perch height and distance to trunk include trunk girth as covariate for arboreal and semi-arboreal species, where *β* is partial coefficient for diel phase. Mean values are based on observed data (in mm). Model for *Sitana marudhamneydhal* is not reported as observations did not show any variation. *Homogeneity of variance assumption violated according to Levene’s test of model residuals

Characteristics	Habitat	Species	Mean Active (SE)	Mean Sleep (SE)	β	SE	df	*p*-value	*R^2^*
*Perch height*	Arboreal	*Salea anamallayana*	1060 (104.93)	1360 (118.44)	0.12	0.06	47	**0.04**	0.06
		*Salea horsfieldii*	1169.62 (99.49)	1095.47 (71.45)	–0.04	0.06	66	0.57	0
	Semi-arboreal	Calotes versicolor*	924.51 (133.49)	987.96 (97.69)	0.18	0.10	49	0.085	0.08
		Monilesaurus rouxii*	984.09 (98.87)	1699.81 (86.07)	0.20	0.07	45	**0.004**	0.39
	Rupicolous	*Psammophilus dorsalis*	816.56 (138.03)	1279.14 (160.69)	0.34	0.19	35	0.825	0.06
	Ground	*Sarada superba*	29.96 (12.18)	0	–0.51	0.27	37	0.072	0.06
		Sitana visiri*	128.5 (31.12)	0	–0.96	0.23	62	**< 0.001**	0.21
		*Sitana marudhamneydhal*	-	-	-	-	-	-	-
*Trunk girth*	Arboreal	*S. anamallayana*	47.58 (4.81)	42.53 (3.35)	–0.05	0.04	48	0.249	0.01
		*S*. *horsfieldii*	64.74 (12.97)	34.45 (2.59)	–0.20	0.06	67	**< 0.001**	0.15
	Semi-arboreal	*C*. *versicolor*	512.14 (74.99)	323.04 (49.4)	–0.20	0.09	50	**0.035**	0.07
		*M*. *rouxii*	1055.95 (180.36)	148.03 (38.74)	–0.90	0.12	46	**< 0.001**	0.54
*Distance to trunk*	Arboreal	*S*. *anamallayana*	308	343.80 (66.63)	–0.10	0.22	47	0.65	0.03
			(48.76)						
		S. horsfieldii*	286.40 (51.99)	290.23 (55.36)	–0.44	0.28	66	0.115	0.03
	Semi-arboreal	*C*. *versicolor*	624.96 (137.63)	502.6 (96.79)	0.11	0.25	49	0.664	0
		*M. rouxii*	30.90 (21.35)	1117.57 (149.92)	2.49	0.31	44	**< 0.001**	0.70

**Table 3:** Chi-square test of proportions for change between diel phases in substrate type, perch type, and perch angle of Agamid lizards. Statistically significant tests (α = 0.05) in bold. Dashes indicate no tests performed due to no variation in observed data

Habitat	Species	Substrate type			Perch type			Perch angle		
		*χ^2^*	*df*	*p-value*	*χ^2^*	*df*	*p-value*	*χ^2^*	*df*	*p-value*
Arboreal	*S. anamallayana*	***-***	***-***	***-***	14.02	2	**0.001**	1.92	2	0.38
	*S. horsfieldii*	***-***	***-***	***-***	4.16	3	0.244	5.42	2	0.06
Semi-arboreal	*C. versicolor*	6.71	2	**0.035**	9.82	3	**0.02**	1.53	2	0.46
	*M. rouxii*	15.38	1	**< 0.001**	36.8	2	**< 0.001**	33.7	2	**< 0.001**
Rupicolous	*P. dorsalis*	2.08	2	0.354	2.08	2	0.354	0.86	1	0.351
Ground	*S. marudhamneydhal*	***-***	***-***	**-**	2.54	1	0.111	-	-	-
	*S. superba*	0.94	1	0.333	9.56	5	0.089	-	-	-
	*S*. *visiri*	56.67	2	**< 0.001**	38.70	2	**< 0.001**	14.39	2	**< 0.001**

## Data Availability

The dataset generated and/or analyzed for the study is available as Online Resource 2.

## Significance

Animals face different ecological conditions and requirements during activity and sleep. One way to adjust for these is by choosing sleep sites that are distinct from active sites, but this prediction is untested in many animals. In this study, we compared the structure of sites used by diurnal lizards during the day vs. the night, in eight species of four types: fully tree-dwelling (arboreal), tree and ground-dwelling (semi-arboreal), rock-dwelling (rupicolous), and ground-dwelling. We find that some sleep site characteristics indeed differ in most species, either in terms of substrate type or perch structure (such as height and girth). For example, both arboreal and semi-arboreal lizards sleep on narrow, flexible perches that likely help them detect and avoid predators through vibrations if they attack. Behaviour studies and conservation of species must therefore account for sleep microhabitats. Significance statementAnimals face different ecological conditions and requirements during activity and sleep. One way to adjust for these is by choosing sleep sites that are distinct from active sites, but this prediction is untested in many animals. In this study, we compared the structure of sites used by diurnal lizards during the day vs. the night, in eight species of four types: fully tree-dwelling (arboreal), tree and ground-dwelling (semi-arboreal), rock-dwelling (rupicolous), and ground-dwelling. We find that some sleep site characteristics indeed differ in most species, either in terms of substrate type or perch structure (such as height and girth). For example, both arboreal and semi-arboreal lizards sleep on narrow, flexible perches that likely help them detect and avoid predators through vibrations if they attack. Behaviour studies and conservation of species must therefore account for sleep microhabitats.
