# Supplementary material for: How distinct are sleep sites from active sites across habitat types in lizards?
Source: Behav Ecol Sociobiol. Author manuscript; Available in PMC 2026 Mar 4. (PMC7618821; doi:10.1007/s00265-026-03693-w)
Supplement: Supplementary Material 1 [file EMS212581-supplement-Supplementary_Material_1.docx]

**How distinct are sleep sites from active sites across habitat types in lizards?**

Nitya Prakash Mohanty^1,2,*^, Anbazhagan Abinesh^1^, Saumitra Dhere^1^, Maria Thaker^1^

^1^Centre for Ecological Sciences, Indian Institute of Science, Bengaluru, India

^2^Département Adaptations du Vivant, UMR 7179, Mécanismes adaptatifs et Évolution (MECADEV) CNRS/Muséum national d'Histoire naturelle, Paris, France

^*^Corresponding author e-mail id: [nitya.mohanty@gmail.com](mailto:nitya.mohanty@gmail.com)

**Online Resource 1**

**Table 1** Sampling locations and effort (sample size, n), and mean body size (snout-vent length) for eight Agamid lizard species in peninsular India. The individuals sampled for each phase are unique.

| **Species** | **n (active)** | **n (sleep)** | **SVL (SD)**  **active** | **SVL (SD)**  **sleep** | **Type** | **Site** | **Habitat description** |
| --- | --- | --- | --- | --- | --- | --- | --- |
| *Salea anamallayana* | 24 | 26 | 8.59  (0.76) | 9.21  (0.84) | Arboreal | Kodaikanal | Plantations of *Eucalyptus grandis* and/or *Acasia mearnsii* adjoining Southern tropical montane wet evergreen forest (Shola), with invasive plants (*Eupatorium glandulosus*, *Parthenium hysterophorus*, and *Lantana camara*) |
| *Salea horsfieldii* | 27 | 42 | 7.2  (0.92) | 6.82  (1.18) | Arboreal | Ooty | Plantations of *Eucalyptus grandis* adjoining Southern tropical montane wet evergreen forest (Shola), with Shola trees and invasive plants (*Cestrum aruntiacum* and *Eupatorium gladulosum*) |
| *Calotes versicolor* | 30 | 25 | 11.9  (1.41) | 9.11  (1.61) | Semi-arboreal | Madurai | Scrub jungle and rocky landscape, adjoining inhabited areas |
| *Monilesaurus rouxii* | 21 | 27 | 6.9  (0.56) | 6.08  (0.84) | Semi-arboreal | Kudremukh | Tropical semi- evergreen forests |
| *Psammophilus dorsalis* | 16 | 21 | 8.98  (2.71) | 10.82  (2) | Rupicolous | Coonoor | Rocky landscape adjoining semi- evergreen vegetation and tea plantation |
| *Sarada superba* | 28 | 11 | 6.59  (0.52) | 6.29  (0.35) | Ground-dwelling | Chalkewadi | Grasslands interspersed with rocks |
| *Sitana marudhamneydhal* | 30 | 30 | 4.41  (0.7) | 4.8  (0.64) | Ground-dwelling | Tirunelveli | Grasslands, thorny shrubs, rocks |
| *Sitana visiri* | 34 | 30 | 4.5  (0.34) | 4.5  (0.33) | Ground-dwelling | Madurai | Shrub jungle, thorny shrubs, rocks |

**Table 2** Principal Component Analysis (PCA) loadings of variables sorted in descending order of absolute value by PC1.

| ***Salea anamallayana*** | **PC1** | **PC2** | **PC3** |
| --- | --- | --- | --- |
| Girth | 0.68 | 0.19 | -0.14 |
| Perch diameter | 0.67 | 0.22 | 0.2 |
| Perch height | -0.2 | 0.69 | 0.58 |
| Distance to main trunk | -0.2 | 0.66 | -0.61 |
| Perch orientation - Horizontal | -0.07 | -0.02 | 0.21 |
| Perch orientation - Vertical | 0.06 | -0.05 | -0.12 |
| Head direction - Inward | -0.05 | 0 | 0.31 |
| Head direction - Perpendicular | 0.03 | -0.02 | -0.04 |
| Head direction - Outward | 0.02 | 0.01 | -0.26 |
| Perch orientation - Angular | 0.01 | 0.08 | -0.09 |
| Substrate - Shrub | 0 | 0 | 0 |
| Substrate - Tree | 0 | 0 | 0 |
| Substrate - Ground | 0 | 0 | 0 |
| Substrate - Rock | 0 | 0 | 0 |
| Substrate - Ground-Under Shrub | 0 | 0 | 0 |

| ***Salea horsfieldii*** | **PC1** | **PC2** | **PC3** |
| --- | --- | --- | --- |
| Girth | 0.7 | 0.03 | 0.17 |
| Perch diameter | 0.69 | -0.15 | 0 |
| Distance to main trunk | 0.14 | 0.64 | -0.71 |
| Perch orientation - Angular | 0.08 | 0.1 | 0.12 |
| Perch orientation - Vertical | -0.08 | -0.17 | -0.04 |
| Perch height | -0.05 | 0.69 | 0.66 |
| Head direction - Outward | -0.01 | -0.17 | 0.06 |
| Head direction - Inward | 0.01 | 0.13 | -0.06 |
| Perch orientation - Horizontal | 0 | 0.07 | -0.08 |
| Head direction - Perpendicular | 0 | 0.04 | 0 |
| Substrate - Shrub | 0 | 0 | 0 |
| Substrate - Ground | 0 | 0 | 0 |
| Substrate - Tree | 0 | 0 | 0 |
| Substrate - Rock | 0 | 0 | 0 |
| Substrate - Ground-Under Shrub | 0 | 0 | 0 |

| ***Monilesaurus rouxii*** | **PC1** | **PC2** | **PC3** |
| --- | --- | --- | --- |
| Perch diameter | 0.5 | 0.4 | 0.2 |
| Distance to main trunk | -0.43 | 0.55 | 0.38 |
| Girth | 0.42 | 0.48 | 0.1 |
| Perch height | -0.41 | 0.39 | -0.67 |
| Perch orientation - Vertical | 0.24 | -0.05 | -0.17 |
| Head direction - Outward | 0.23 | -0.14 | -0.23 |
| Head direction - Inward | -0.21 | 0.12 | 0.25 |
| Substrate - Shrub | -0.15 | -0.24 | 0.31 |
| Substrate - Tree | 0.15 | 0.24 | -0.31 |
| Perch orientation - Horizontal | -0.13 | 0.01 | 0.14 |
| Perch orientation - Angular | -0.11 | 0.05 | 0.02 |
| Head direction - Perpendicular | -0.01 | 0.02 | -0.02 |
| Substrate - Ground | 0 | 0 | 0 |
| Substrate - Rock | 0 | 0 | 0 |
| Substrate - Ground-Under Shrub | 0 | 0 | 0 |

| ***Calotes versicolor*** | **PC1** | **PC2** | **PC3** |
| --- | --- | --- | --- |
| Girth | 0.55 | -0.36 | -0.13 |
| Perch diameter | 0.48 | -0.51 | 0.29 |
| Perch height | 0.47 | 0.48 | -0.49 |
| Distance to main trunk | 0.4 | 0.57 | 0.62 |
| Substrate - Shrub | -0.2 | 0 | 0.17 |
| Substrate - Tree | 0.2 | 0 | -0.17 |
| Head direction - Inward | -0.07 | 0.05 | 0.18 |
| Head direction - Outward | 0.05 | -0.1 | -0.24 |
| Perch orientation - Vertical | -0.02 | -0.14 | 0 |
| Head direction - Perpendicular | 0.02 | 0.05 | 0.06 |
| Perch orientation - Angular | 0.02 | -0.02 | 0.25 |
| Perch orientation - Horizontal | 0.01 | 0.16 | -0.25 |
| Substrate - Ground | 0 | 0 | 0 |
| Substrate - Rock | 0 | 0 | 0 |
| Substrate - Ground-Under Shrub | 0 | 0 | 0 |

| ***Psammophilus dorsalis*** | **PC1** | **PC2** | **PC3** |
| --- | --- | --- | --- |
| Perch Height | 0.98 | -0.1 | -0.14 |
| Perch Orientation - Horizontal | -0.09 | -0.25 | -0.45 |
| Perch orientation - Vertical | 0.09 | 0.25 | 0.45 |
| Substrate - Rock | 0.06 | 0.25 | 0.35 |
| Head direction - Upward | 0.06 | 0.81 | -0.23 |
| Head direction - Angular | 0.06 | -0.32 | 0.54 |
| Substrate - Ground | -0.05 | -0.15 | -0.31 |
| Head direction - Downward | -0.02 | -0.06 | 0.12 |
| Substrate - Shrub | -0.01 | -0.1 | -0.04 |
| Head direction - Perpendicular | 0.01 | -0.08 | 0.06 |
| Substrate - Tree | 0 | 0 | 0 |
| Substrate – Ground-Under Shrub | 0 | 0 | 0 |
| Perch orientation - Angular | 0 | 0 | 0 |
| Head direction - Outward | 0 | 0 | 0 |
| Head direction - Inward | 0 | 0 | 0 |

| ***Sarada superba*** | **PC1** | **PC2** | **PC3** |
| --- | --- | --- | --- |
| Perch Height | 0.96 | 0.29 | 0 |
| Substrate - Ground | -0.21 | 0.68 | 0 |
| Substrate - Rock | 0.21 | -0.68 | 0 |
| Substrate - Shrub | 0 | 0 | 0 |
| Substrate - Tree | 0 | 0 | 0 |
| Substrate - Ground-Under Shrub | 0 | 0 | 0 |
| Perch orientation- Angular | 0 | 0 | 0 |
| Perch orientation - Horizontal | 0 | 0 | 0 |
| Perch orientation - Vertical | 0 | 0 | 0 |
| Head direction - Upward | 0 | 0 | 0 |

| ***Sitana visiri*** | **PC1** | **PC2** | **PC3** |
| --- | --- | --- | --- |
| Perch height | 0.81 | 0.03 | -0.24 |
| Substrate - Shrub | 0.32 | 0.01 | 0.35 |
| Head direction - Outward | 0.32 | 0.01 | 0.35 |
| Perch orientation - Vertical | 0.29 | 0.01 | -0.36 |
| Substrate - Ground-Under Shrub | -0.18 | 0.7 | -0.17 |
| Substrate - Ground | -0.14 | -0.71 | -0.18 |
| Perch orientation - Angular | 0.03 | 0 | 0.71 |
| Substrate - Tree | 0 | 0 | 0 |
| Substrate - Rock | 0 | 0 | 0 |
| Perch orientation - Horizontal | 0 | 0 | 0 |
| Head direction - Inward | 0 | 0 | 0 |
| Head direction –Perpendicular | 0 | 0 | 0 |

**Table 3** Chi-square test of proportions for changes in head direction between diel phases. For semi-arboreal and arboreal lizards on plants head direction was categorized as ‘inwards’, ‘outwards’, ‘perpendicular’, whereas for *Psammophilus dorsalis* on rocks, it was ‘upward’, ‘angular-upward’, ‘perpendicular’, and ‘downward’. Statistically significant tests (α = 0.05) in bold.

| **Species** | *χ^2^* | *df* | *p-value* |
| --- | --- | --- | --- |
| *S*. *anamallayana* | 5.29 | 2 | 0.07 |
| *S*. *horsfieldii* | 4.88 | 2 | 0.09 |
| *C*. *versicolor* | 13.3 | 2 | **<0.001** |
| *M*. *rouxii* | 30.57 | 2 | **<0.001** |
| *P. dorsalis* | 4.95 | 3 | 0.174 |

**
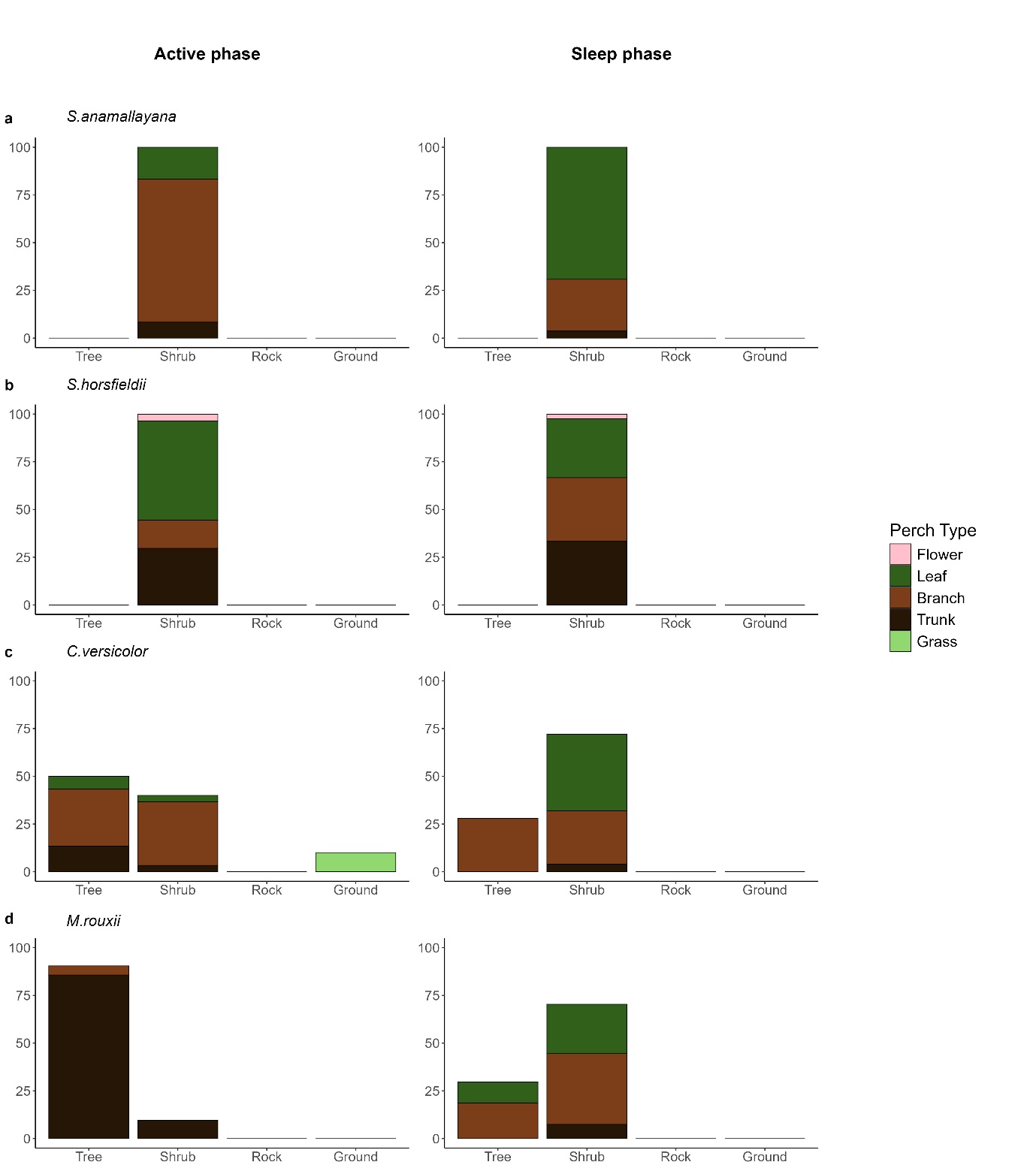
**

**Fig. 1** Use of substrate types and perch types by arboreal (*Salea anamallayana*, *Salea horsfieldii*) and semi-arboreal (*Calotes versicolor*, *Monilesaurus rouxii*) Agamid lizards during active (plots on left) and sleep phases (plots on right). Substrate types within each phase sum to 100% and perch types within each substrate type sum to 100%.

**
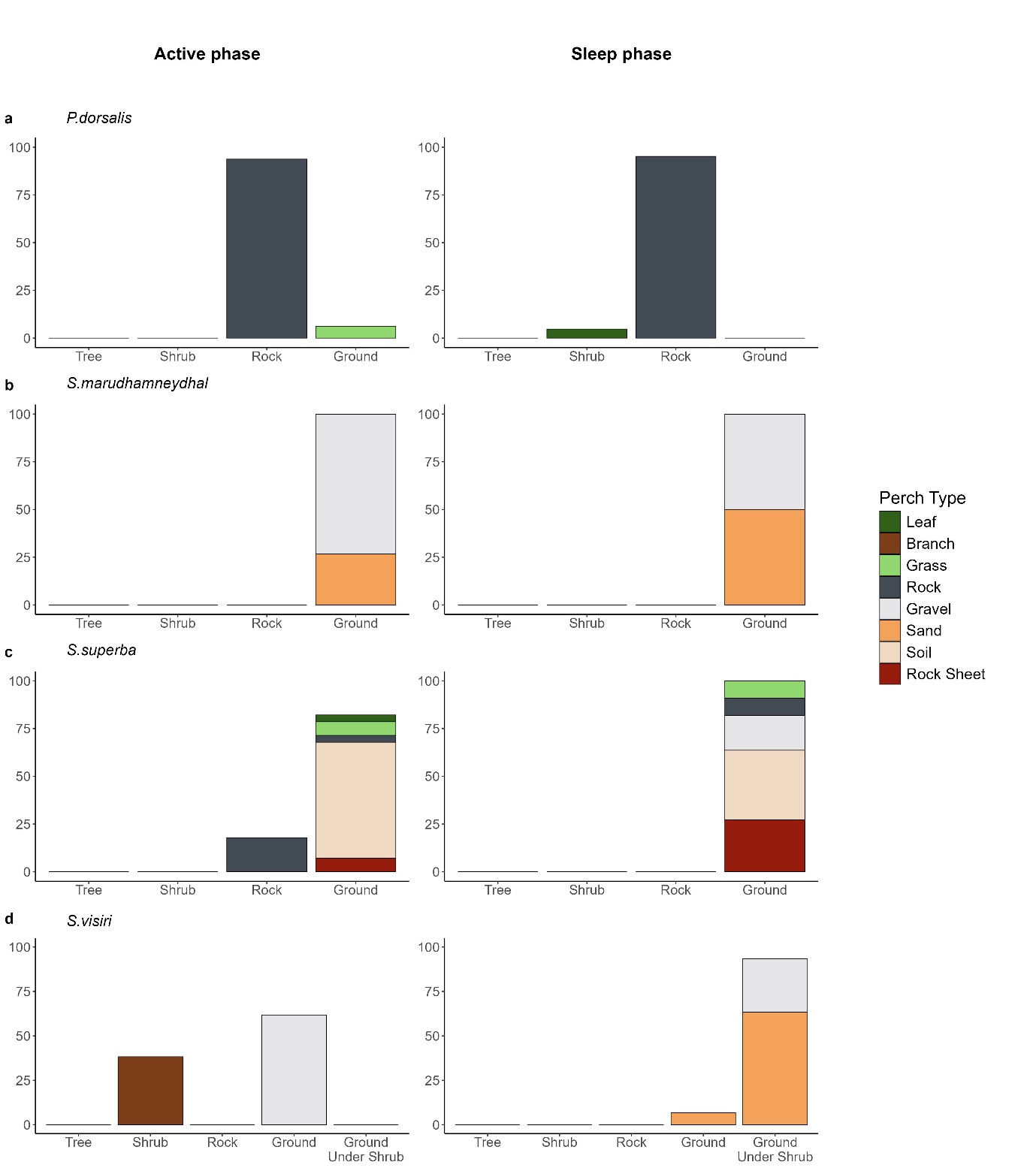
**

**Fig. 2** Use of substrate types and perch types by rupiculous (*Psammophilus dorsalis*) and ground-dwelling (*Sarada superba*, *Sitana marudhamneydhal*, *Sitana visiri*) Agamid lizards during active (plots on left) and sleep phases (plots on right). Substrate types within each phase sum to 100% and perch types within each substrate type sum to 100%.
